# Genome-wide DNA methylation changes with age in disease-free human skeletal muscle

**DOI:** 10.1111/acel.12180

**Published:** 2013-12-02

**Authors:** Artem Zykovich, Alan Hubbard, James M Flynn, Mark Tarnopolsky, Mario F Fraga, Chad Kerksick, Dan Ogborn, Lauren MacNeil, Sean D Mooney, Simon Melov

**Affiliations:** 1 Buck Institute for Research on Aging8001 Redwood Blvd, Novato, CA, 94945, USA; 2 Division of Biostatistics, School of Public Health, University of California101 Haviland Hall, MC 7358, Berkeley, CA, 94720, USA; 3 Neuromuscular and Neurometabolic Unit, Rm. 2H26, McMaster Children’s Hospital, McMaster University Medical Center1200 Main St. W., Hamilton, Ontario, Canada, L8N 3Z5; 4 Cancer Epigenetics Laboratory, Department of Immunology and Oncology, Centro Nacional de Biotecnología/CNB-CSIC, Instituto Universitario de Oncología del Principado de Asturias (IUOPA), HUCA, Universidad de Oviedo33006, Oviedo, Spain; 5 Department of Health, Exercise and Sport Sciences, University of New MexicoAlbuquerque, NM, 87109, USA

**Keywords:** DNA methylation, skeletal muscle, human aging, epigenome, genomics, postmitotic

## Abstract

A decline in skeletal muscle mass and function with aging is well recognized, but remains poorly characterized at the molecular level. Here, we report for the first time a genome-wide study of DNA methylation dynamics in skeletal muscle of healthy male individuals during normal human aging. We predominantly observed hypermethylation throughout the genome within the aged group as compared to the young subjects. Differentially methylated CpG (dmCpG) nucleotides tend to arise intragenically and are underrepresented in promoters and are overrepresented in the middle and 3′ end of genes. The intragenic methylation changes are overrepresented in genes that guide the formation of the junction of the motor neuron and myofibers. We report a low level of correlation of gene expression from previous studies of aged muscle with our current analysis of DNA methylation status. For those genes that had both changes in methylation and gene expression with age, we observed a reverse correlation, with the exception of intragenic hypermethylated genes that were correlated with an increased gene expression. We suggest that a minimal number of dmCpG sites or select sites are required to be altered in order to correlate with gene expression changes. Finally, we identified 500 dmCpG sites that perform well in discriminating young from old samples. Our findings highlight epigenetic links between aging postmitotic skeletal muscle and DNA methylation.

## Introduction

Aging is accompanied by a reduction in muscle mass and function, which is commonly referred to as sarcopenia and leads to decreased mobility (Marzetti *et al*., 2009), with an estimated cost to the US economy of some 18.5 billion a year (Janssen *et al*., 2004). Although numerous different biochemical pathways have been linked with sarcopenia, the mechanisms that drive the development of this important age-related disorder remain to be uncovered. There have been several studies focused at the molecular level that have reported changes in skeletal muscle with age and exercise. However, these studies have primarily focused on the quantitation of RNA abundance (Melov *et al*., 2007; Drummond *et al*., 2011) or have not stringently controlled for confounding effects such as activity level, comorbidities, medications, and timing of the last exercise bout. In addition to measuring gene expression, the epigenetic modification of DNA cytosine plays important roles in many processes including differentiation and cellular senescence (Berdasco & Esteller, 2011). Aging cells, different tissue types as well as a variety of human diseases possess their own distinct DNA methylation profiles (Fernandez *et al*., 2012). Dysregulation of DNA methylation has also been reported with age-related disorders such as Alzheimer’s disease (Irier & Jin, 2012) and cancer (Lopez-Serra & Esteller, 2012).

Changes in DNA methylation with aging have also been reported in a select few tissues of human beings, as well as in a number of other species (Calvanese *et al*., 2009). One of the initial studies reported a decline in methylation in a number of aged mouse tissues (Wilson *et al*., 1987). Another early study observed promoter hypermethylation with age in human colorectal mucosa and argued that aging is a major contributing factor to hypermethylation in cancer of this tissue (Ahuja *et al*., 1998). A longitudinal study of DNA methylation in human blood cells reported that DNA methylation changes with age (Bjornsson *et al*., 2008). A recent report compared global hypomethylation of DNA from blood in centenarians with middle-aged individuals and newborns (Heyn *et al*., 2012). However, in general, molecular studies on aged tissues from well-characterized cohorts absent of potentially confounding age-related disorders are relatively uncommon. The majority of DNA methylation studies on aging have been carried out on blood comprised of multiple cell types. No studies have as of yet examined postmitotic skeletal muscle for epigenetic changes between young and old individuals. A minor surgical procedure is required to obtain such tissues, contributing to the difficulty in carrying out such studies.

Here, we report for the first time a genome-wide study of DNA methylation dynamics in a postmitotic tissue – skeletal muscle taken from healthy older adults compared to young adults under controlled conditions at over 480 000 sites throughout the genome. We report a predominant pattern of hypermethylation in the DNA of aging skeletal muscle. Surprisingly, the locations of these dmCpG sites are underrepresented in promoter regions and are overrepresented in the middle and 3′ ends of genes. These epigenetic changes could be very significant to the biology of aging muscle as recent studies have suggested that age-related loss of muscle mass is not due to the loss of muscle cells, but a reduction in type II fiber size (Nilwik *et al*., 2013). In comparing our data with prior gene expression studies, we did not observe a general correlation of DNA methylation with differentially expressed genes in aged human skeletal muscle. Finally, using a novel approach to identify dmCpGs associated with aging, we have identified over 500 methylated sites that separate younger subjects from older subjects in this study.

## Results

### Distribution of differentially methylated CpG sites

**Figure 1:**
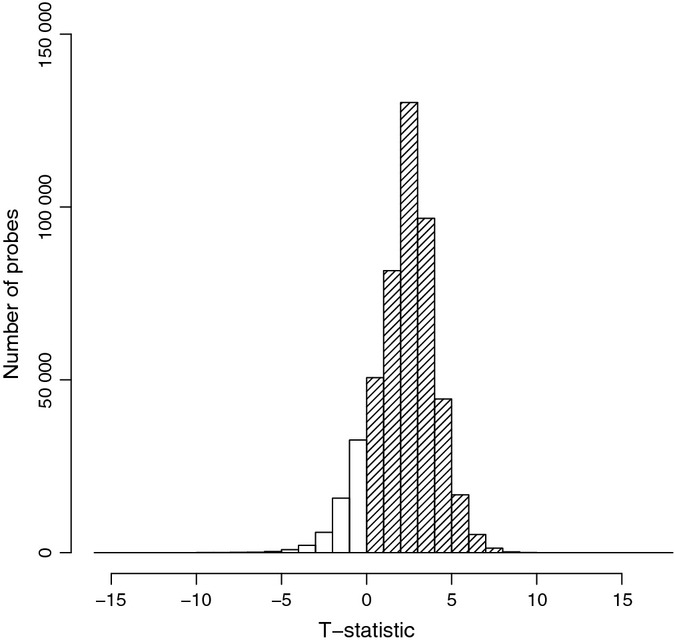
Changes in methylation status with age. Distribution of the *t*-statistic shows a significant hypermethylation of DNA derived from skeletal muscle of older versus younger adults (distribution is shifted to the right). *Y*-axis: number of CpG probes; *x*-axis: *t*-statistic. Filled columns are hypermethylated with age, and uncolored columns hypomethylated with age.

**Figure 2:**
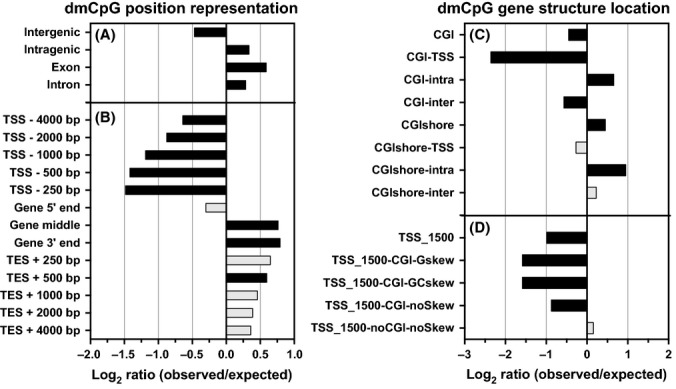
Distribution of dmCpG. Log(2) ratio of observed [fraction of dmCpG from all CpG sites on the microarrays (aCpG) in a region] to expected (fraction of dmCpG from aCpG in genome) for a region. Black color indicates a significant overrepresentation (*P* < 0.05) of dmCpG. The positional categories of dmCpG contain the location of dmCpG sites in relation to specific sequence features in the genome. The number following TSS/TES refers to the number of nucleotides upstream and downstream of TSS/TES. A detailed regional description and the breakdown of the hyper- and hypomethylated site observed/expected ratios can be found in Table S12 (Supporting Information) and Table S2.

We have studied DNA methylation dynamics in skeletal muscle taken from 24 healthy older male adults (age range: 68–89 years) compared to 24 young male adults (age range: 18–27 years) under controlled conditions at 485 577 sites throughout the genome (Illumina 450 K methylation arrays). We found an overall global trend of genomic hypermethylation in human skeletal muscle between the young and old groups (Fig. 1). We identified 5963 CpG sites that are differentially methylated between the two groups (dmCpG). Of these, 92% (5518 dmCpG sites) were hypermethylated in the older subjects, while the remaining 8% were hypomethylated (Table S1, Supporting Information). We observed that the distribution of dmCpG sites was relatively even over all chromosomes, with exceptions of a significant overrepresentation of dmCpGs in chromosomes 16, 17 and a relative underrepresentation in the X chromosome (Table S2, Supporting Information). For both hyper- and hypomethylated dmCpG sites, there was a significant enrichment within a gene and there was an underrepresentation outside of genes (Fig. 2A, Table S2). In order to further refine positional information about dmCpGs with age relative to gene structure, we segmented genes into the following structural regions: the 5′ region, the ‘central’ region, the 3′ region, and regions around the transcription start, and end sites (TSS and TES). DmCpG sites within genes could be divided into two broad regions – those that were underrepresented near the 5′ regions of genes and those that were enriched in the central and 3′ regions (Fig. 2B, Table S2).

CpG islands (CGI) are present in 60% of gene promoters in the 450 K chip, and methylation deregulation in CGI overlapping the promoter has often been linked with cancer (Draht *et al*., 2012). Other relevant features of note in the methylated DNA of cancer are increases in the inter- and intragenic CGI (Deaton *et al*., 2011) and CGI shores (Irizarry *et al*., 2009). In order to examine whether a similar phenomenon arises in aged human skeletal muscle, we tested the association between methylation and age in CGI and CGI shores (2000 base pairs upstream and downstream of a CGI) that overlap with promoters, and intra-/intergenic regions. We determined that in aging human skeletal muscle, there is an overall enrichment of dmCpG sites in CGI shores and there is a decrease in CGI, except for CGI within a gene (Fig. 2C, Table S2). All data including methylation values/subject, and methylated vs. unmethylated probe data, are deposited in GEO and are accessible via the GEO number GSE50498.

Recently, it was determined that an R-loop (a three-stranded nucleic acid structure formed due to biased strand distribution of guanines and cytosines, or GC-skew) negatively correlates with the corresponding promoter methylation level (Ginno *et al*., 2012). For each promoter region (−1500 +1500 bp around TSS), we defined those that have or do not have CGI or GC-skew, or both. We identified that there was an underrepresentation of dmCpG sites in all promoters, unless it is a promoter with no CGI and nonskew (Fig. 2D, Table S2). Ginno *et al*. reported that promoters with CGI and positive GC-skew are the most protected from methylation, while as promoters with weak CGI and weak GC-skew showed little to no protection. Concordant with these results, we report that less protected promoters in DNA of aged skeletal muscle tend to acquire more methylation than the younger tissue.

As a final analysis of the positional information of the dmCpG sites within aged skeletal muscle, we performed an analysis for possible enrichment of differential methylation of transcriptional regulator-binding sites. Using the ENCODE ChIP-Seq Significance Tool, we were able to probe existing datasets for enriched transcription factors within our gene list (Auerbach *et al*., 2013). We used all skeletal muscle cell lines (Hsmm, Hsmmt) available within the ENCODE project and then analyzed gene sets from our data that have at least 2, 4, or 8 dmCpG within their gene boundaries. In all three cases, the only significantly enriched motif (*P* < 0.01) was the transcription factor CTCF (Table S3, Supporting Information).

### Genes with altered methylation status within aged muscle tissue

**Figure 3:**
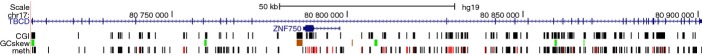
Schematic distribution of CpG islands (CGI), GC-skew, and CpG sites within TBCD gene. This figure was generated using the Genome Browser (Kent *et al*., 2002). GC-skew track: Gskew on positive strand marked with green; Gskew on negative strand marked with brown. Methylation (meth) track: CpG sites that are present on the arrays are colored black; dmCpG sites are colored red.

We identified 2114 genes with at least one dmCpG site located intragenically (Table S4, Supporting Information) in DNA from aged skeletal muscle. The location of these dmCpG sites may be significant, as methylation changes in aged individuals may affect gene function (Maunakea *et al*., 2010) and consequently muscle function. We observed that the number of dmCpG sites for a gene was not dependent on the total number of CpG sites present on the microarray chip for the same gene (Pearson’s correlation = 0.55, and Fig. S1, Supporting Information). One example of a gene with dmCpG that is affected by aging is the tubulin-folding cofactor D (TBCD) gene (Fig. 3). Tubulin-folding cofactor D has the highest number of intragenic dmCpG sites (46 distinct sites, which is 13.2% of the total number of CpG sites present for the gene). Of these, 45 of them were hypermethylated in the aged group compared to the young muscle tissue, a significant enrichment in the older age group. As an important component of microtubules, TBCD plays a key role in all cell types, and disruption of microtubule pathways was previously reported to affect motor neuron function (Molon *et al*., 2004). Another interesting example of dmCpG within the older age group is the gene for pericentrin, with three dmCpG sites. We report that all three of these sites were hypomethylated with age (4.1% of total), and expression of this gene has also been reported to be increased with age (Melov *et al*., 2007). Like TBCD, the pericentrin protein is a part of the cytoskeleton, and it plays an important role in microtubule assembly and reorganization during muscle differentiation (Bugnard *et al*., 2005).

### Ontology and pathway enrichment analysis

In order to better understand the biological significance of dmCpG changes in aged skeletal muscle, we applied STOP, an ontology enrichment tool (Wittkop *et al*., 2013), to genes with two or more intragenic dmCpG sites. Only one enriched term was found from cell type ontology analysis, the term ‘muscle cell’ (*P* = 0.0004). This suggests that the dmCpG sites we identified within the aged group are relevant to muscle tissue. There were 54 enriched terms with a *P* < 0.01 (Table S5, Supporting Information). This list includes muscle-related terms (two terms) and signal transduction/intracellular transport (25 terms), such as axon (*P* = 2.05E-07) and axon guidance (*P* = 2.11E-07), which may be related to muscle innervation and aging. A decline of function in the neuromuscular junction has long been thought to contribute to the decline of muscle mass with age (Bütikofer *et al*., 2011). Actin cytoskeleton (*P* = 3.89E-05), cytoskeleton (*P* = 0.0003), and cytoskeleton organization (*P* = 0.001) are all linked to cytoskeleton function, and the latter plays an important role in proper muscle contractile function (Berthier & Blaineau, 1997). Other significantly identified terms could be put together into the cell adhesion/motility group (six terms), including plasma membrane (*P* = 3.23E-08), homophilic cell adhesion (*P* = 8.43E-08), and cell adhesion (*P* = 1.70E-05), with cell adhesion being associated with fiber degeneration (Campbell, 1995). Additionally, we observed several terms that could be gathered into a growth and differentiation group (six terms), ion binding (two terms), broad cellular function (11 terms), and two terms that stand separately.

We further expanded our analysis by examining the overrepresentation of canonical genetic pathways specific to muscle tissue using ingenuity pathway analysis (www.ingenuity.com) (Table S6, Supporting Information). This analysis further expanded on the STOP ontology evaluation by identifying ‘axon guidance signaling’ as the top pathway within the dmCpG dataset (*P* = 6.16E-10) with 50 of 216 pathway members (23.1%) differentially methylated in at least one intragenic site (Table S7 and Fig. S2, Supporting Information).

A recent report utilized genes identified as having dmCpGs with age to examine stem cell differentiation pathways (West *et al*., 2013). We compared our results from postmitotic skeletal muscle to their findings of the best age predictor in whole blood – the UTF1 gene, which is also hypermethylated with age. We did not find any dmCpG sites within or around this gene in DNA obtained from skeletal muscle. We also did not observe dmCpG sites in/around the other age predictors WNT3A, SFRP1, FZD2, or FZD9 previously reported for whole blood. Another study of DNA methylation of whole blood and aging found a hypermethylation of bivalent chromatin domains with increasing age (Rakyan *et al*., 2010). The comparison of the sites from that study and our current study of skeletal muscle found that there was a significant overlap in genes that contain both dmCpG sites in bivalent chromatin domains (hypergeometric test, *P* = 7E-33).

### Comparison of DNA methylation state with gene expression

Next, we compared the differentially methylated sites identified in DNA from aged skeletal muscle with a previously reported study on gene expression using a similar experimental design, which was focused on healthy older adults absent age-related disease (Melov *et al*., 2007). We could not carry out gene expression and genome-wide methylation on all samples, as the amount of material obtained via needle biopsy was limited at the time we carried out the study. We wished to determine whether there was any relationship between gene expression in healthy aged skeletal muscle and the level of DNA methylation. We identified suites of genes that have a minimum number of differentially methylated CpG sites (1, 2, 4, 8, and 16) within specific regions, for example intragenic, 5′ end, or CGI-TSS. If a gene has only hypermethylated or hypomethylated sites, it was designated as such. We then calculated the number of genes from either hypo- or hypermethylated groups that overlap with genes that were previously reported to be overexpressed or underexpressed within aged muscle tissue, as well as genes that did not change in expression between young and old subjects. We determined that the majority of genes with altered methylation in DNA from healthy aged human skeletal muscle are characterized by unchanged gene expression within aged tissue (Table S8, Supporting Information).

**Figure 4:**
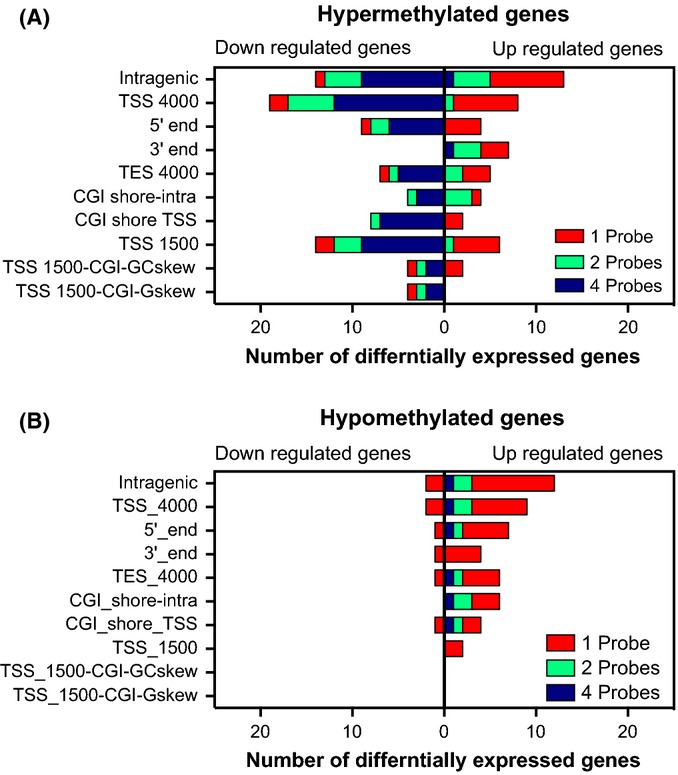
Correspondence of hyper- and hypo-dmCpG in genes with changes in gene expression. Number of genes (*x*-axis) that have a minimal number of hypermethylated (A) or hypomethylated dmCpG (B). The data are displayed as a stacked bar chart for one probe (red), two probes (green), or four probes (blue) for each genome position category. The expression change in aged tissue is indicated as bars. Left of axis is down-regulated and bars to the right are up-regulated. Detailed description of genome position categories is described in Table S12.

We then focused only on genes that were significantly altered in the aged group as compared to the young group, and calculated how changes in gene expression correlate with changes in DNA methylation. We found that a majority of hypomethylated genes had an increase in gene expression with age, while hypermethylated genes displayed a reduction in gene expression (Fig. 4). Only for dmCpG sites in the 3′ ends of genes did we observe a positive concordance between hypermethylation and up-regulation of gene expression, which has also been reported by other groups in differing biological contexts (Hellman & Chess, 2007; Feng *et al*., 2010). These correlations were especially strong with increasing numbers of dmCpG sites within a gene or gene region. These results suggest that a minimal number of dmCpG sites or select sites are required to be altered in order to inversely modulate the gene expression in aging skeletal muscle. Similar results were also reported for different tissues in another methylation study (Weber *et al*., 2007).

### Differential methylation sites with age

**Figure 5:**
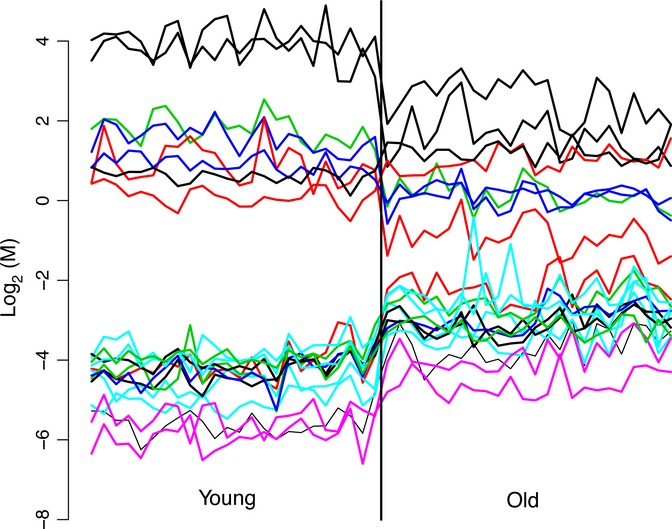
Methylation level of 21 CpG probes with M-values ranked by age. Individual methylation levels of 21 CpG sites that distinguish young from old subjects.

From all dmCpG in DNA from aged skeletal muscle, there are 500 high probability predicting CpG sites (Table S9, Supporting Information) and 21 CpG sites that are universally changed in either the young or old age groups’ CpG predictors (i.e., either all increased in all old samples, and all decreased in all young samples, or the inverse) (Fig. 5). We used a procedure in which cross-validation was used to estimate the misclassification rate. It also incorporates simple stepwise constant functions as candidates and will result in no misclassified samples (see methods for a more detailed description). A majority of these sites are increased in methylation within the aged tissue, although some do decrease. Any reasonable prediction procedure that looks for simple cutoffs using even one probe comparing young samples to old samples would have found probes that completely cross-validated (unbiased) in terms of classifying young from old in 21 of the sites, and nearly completely separated young from old for at least 500. Three probes of these 21 sites are excellent at distinguishing young from old, and nine of 500 of these excellent/good predictors were also determined as age-predicting markers by a recent study on DNA methylation in blood (Hannum *et al*., 2012) (Table S10, Supporting Information). However, we stress that such sites would need to be validated in a completely independent population in order to verify their biological significance, although it remains statistically highly unlikely that these 21 probes are differentially methylated between the two age groups by chance alone.

## Discussion

We report for the first time a genome-wide survey of DNA methylation status of skeletal muscle DNA from healthy younger versus older male individuals. We determined that differentially methylated CpG nucleotides predominantly arise intragenically and are underrepresented in promoters and are overrepresented in the middle and 3′ end of genes. We also identified a number of sites that discriminated with high confidence the younger subjects from older subjects.

Historically, it has long been known that DNA methylation changes with age to varying extents, dependent upon the sites being assayed within the genome, species, tissue, or cell (Calvanese *et al*., 2009). Several recent studies have reported genome-wide increases in DNA hypermethylation with age in cells from whole blood or skin (Grönniger *et al*., 2010; Rakyan *et al*., 2010; Bell *et al*., 2012), saliva (Bocklandt *et al*., 2011), and brain (Hernandez *et al*., 2011). Other studies have reported substantial decreases in DNA methylation with age in blood (Teschendorff *et al*., 2010; Heyn *et al*., 2012). These genome-wide studies have used a variety of approaches to cover the genome to a greater or lesser extent, and true genome-wide coverage at the level of the single base is fast approaching. We have compared the dmCpG sites we identified in postmitotic human skeletal muscle to the reported dmCpG sites from six other studies of different tissues. We did not find any common dmCpG site with age in skeletal muscle (this study) and brain (Hernandez *et al*., 2011). With four of these (Rakyan *et al*., 2010; Teschendorff *et al*., 2010; Bocklandt *et al*., 2011; Bell *et al*., 2012), there were only 2–6 dmCpG sites in common (Table S11, Supporting Information). We identified 141 concordant dmCpG sites between aged skeletal muscle and blood (Heyn *et al*., 2012) (Fig. S11, Supporting Information). The technology for identifying dmCpG sites has rapidly evolved over the last few years, progressing from relatively low-throughput assessment of single sites of interest to more than 450 thousand (450 K) sites throughout the genome. We and others (Heyn *et al*., 2012) employed the 450 K Illumina array technology to identify differentially methylated sites with age, while other studies (Rakyan *et al*., 2010; Teschendorff *et al*., 2010; Bocklandt *et al*., 2011; Bell *et al*., 2012) have used the smaller 27 K Illumina platform, surveying < 6% of the total sites present on the 450 K chip. The difference in the number of methylation sites being surveyed between the 27 K and 450 K formats could be a major contributory factor with regard to a lack of concordance between studies, because only three CpG sites from 141 dmCpGs we identified were also present on the smaller format 27 K Illumina array chip. Interestingly, one CpG site was differentially methylated with age in three studies (including this study), another two CpG sites in four studies, and one CpG in five studies. Each of the methylation sites of interest is located intragenically close to the 5′ end of four genes. The genes containing these CpG sites are CELF6, NHLRC1, CECR6, and INSM2. There is little functional information known about CECR6 or INSM2; however, NHLRC1 encodes the subunit E3 ubiquitin ligase and is associated with Lafora epilepsy (Salar *et al*., 2012). CELF6 is a member of CELF protein family, which carries an RNA-binding domain.

For genes with two or more intragenic dmCpG sites identified in DNA from aged skeletal muscle, we have identified several associated gene ontology terms and pathways which were enriched within the aged group versus the young subjects, including ‘axon guidance signaling’. Differential methylation changes in this pathway were used as a focus to identify how epigenetic changes during aging could potentially relate to the well-known loss of skeletal muscle function with increasing age. Motor unit loss (denervation) occurs with aging and is a major contributory factor in sarcopenia (Doherty *et al*., 1993). Therefore, regulation and signaling at the interphase between the nervous system and the musculature is of critical importance during aging to guide the motor neuron to the muscle fiber. Within the ‘axon guidance pathway’ ontology, the gene with the highest number of intragenic dmCpG sites was the NFATC1 (nuclear factor of activated T cells, cytoplasmic 1 gene). This gene contained 17 dmCpG sites in aged skeletal muscle: 16 hypermethylated and one hypomethylated (Table S7). NFATC1 has an established role in transcriptional regulation of skeletal muscle in direct response to electrical stimulation via calcium/calmodulin signaling (McCullagh *et al*., 2004; Rana *et al*., 2008). NFATC1 regulates both signaling at the neuromuscular junction and acts as a nuclear transcriptional regulator of muscle fibers relating information about the contractile electrical impulses entering the cell via motor neurons (Salanova *et al*., 2011). Methylation changes in the DNA for this gene and others within the axon guidance signaling pathway may alter transcriptional events leading to the loss of proper reinnervation at the neuromuscular junction during muscle turnover. The loss of this plasticity mechanism has been shown to be a critical step preceding muscle wasting in both animal models and humans (Lauretani *et al*., 2006; Chai *et al*., 2011; Jang & Van Remmen, 2011). Therefore, the intragenic differential methylation data we identified in DNA from aged skeletal muscle provide potential candidate genes for investigating the role of denervation of the neuromuscular junction loss prior to age-related muscle loss and sarcopenia. This provides a potential functional linkage between epigenetic changes in aged muscle tissue and loss of muscle mass.

In aging human skeletal muscle, we identified a strong preference for the dmCpG sites to localize within a gene, and in the central and 3′ end regions of genes, but surprisingly no preference for the promoter. Some studies have suggested that intragenic DNA methylation could regulate alternative splicing (Sati *et al*., 2012) and may be involved in the regulation of alternative splicing in differentiation and cancer (Irizarry *et al*., 2009). These sites may be also important for gene regulation as they can potentially modulate alternative promoter activity (Maunakea *et al*., 2010). We also identified similar results within CGI, where dmCpGs are overrepresented in intragenic CGI and underrepresented in CGI that overlap the transcriptional start site. Another possible reason for the preferential distribution of dmCpG within DNA of aging skeletal muscle may relate to a potential protective mechanism, where some areas of the genome are more vulnerable to differential methylation than others. Partial evidence for this hypothesis is provided by the observation that promoters without CGI were overrepresented with dmCpG compared to the CGI promoters. It has been previously reported that weak CGI promoters tend to be de novo methylated preferentially in somatic cells during differentiation (Weber *et al*., 2007). Promoters with CGI and positive GC-skew are more protected from methylation and de novo methylation; CGI and GC-skew promoters, however, are less protected from methylation; and finally, promoters with weak CGI and week GC-skew showed little to no protection (Ginno *et al*., 2012). The overall conclusions of these prior studies on targeted protection are concordant with the observations we report here on DNA from aging human skeletal muscle, where we found less than expected dmCpG events in CGI-positive GC-skew promoters compared to promoters without CGI and CG-skew.

A potential issue with the biopsy procedure used in our study is tissue heterogeneity between biopsies, which may impact the resultant molecular signatures. There are other cell types in muscle that might contaminate muscle-specific methylation signatures. Such sample variation may influence the results. However, it was previously demonstrated that there is remarkably little technical variation between discrete biopsies sampled from the right and left leg of the same individual with regard to fiber type differences, gene expression as assessed by microarray, fiber size, or even strength (Tarnopolsky *et al*., 2007).

In summary, we describe for the first time a genome-wide DNA methylation survey of a postmitotic tissue between young and old healthy human skeletal muscle. We also determined that there were several hundred robust dmCpGs that discriminated younger males from older males. Ontology analysis of the terms associated with dmCpGs provided several interesting hints of a potential role for epigenetic changes in the neuromuscular junction during aging. Resistance exercise can reverse specific aspects of the gene expression changes in aging to more youthful levels (Melov *et al*., 2007). Acute endurance exercise has also been shown to decrease promoter methylation in human skeletal muscle (Barrès *et al*., 2012). Collectively, this prior work in combination with the data we report here shows a dynamic inter-relationship between DNA methylation, gene expression, age, and exercise. With exercise, gene expression and DNA methylation can both be ‘reversed’, emphasizing the importance of determining the mechanisms of the beneficial effects of exercise. Future studies will be needed to determine whether resistance exercise alters DNA methylation and can reverse the age-specific methylation profiles we report here.

## Experimental procedures

All methods and experimental procedures are available in online supplement.

## Supporting Information

**Fig. S1** Numbers of CpGs present on array in a gene and dmCpG site for same gene.

**Fig. S2** Ingenuity canonical axon guidance pathway.

**Fig. S3** dmCpG sites that are common for this study and Heyn *et al*.

**Table S1** List of differentially methylated CpG probes with age.

**Table S2** Distribution of dmCpG.

**Table S3** ENCODE ChIP-Seq significance tool results.

**Table S4** Genes with at least one intragenic dmCpG site.

**Table S5** Ontology enrichment analysis.

**Table S6** Muscle-specific canonical pathway analysis.

**Table S7** Differential methylation of axon guidance genes.

**Table S8** Correspondence of intragenic differential methylation to gene expression.

**Table S9** List of CpG sites that discriminate young from old samples.

**Table S10** List of overlapping CpG predictors of biological age with Hannum *et al*. study.

**Table S11** Number of overlapping dmCpG sites with other studies.

**Table S12** Description of genomic regions.

**Table S13** Description of human subjects.

**Table S14** Illumina 450 K data quality control report.
