# Supplementary figures and images for: Genome-wide DNA methylation changes with age in disease-free human skeletal muscle
Source: Aging Cell. 2013 Dec 2;13(2):360–6. doi: 10.1111/acel.12180 (PMC3954952; doi:10.1111/acel.12180)

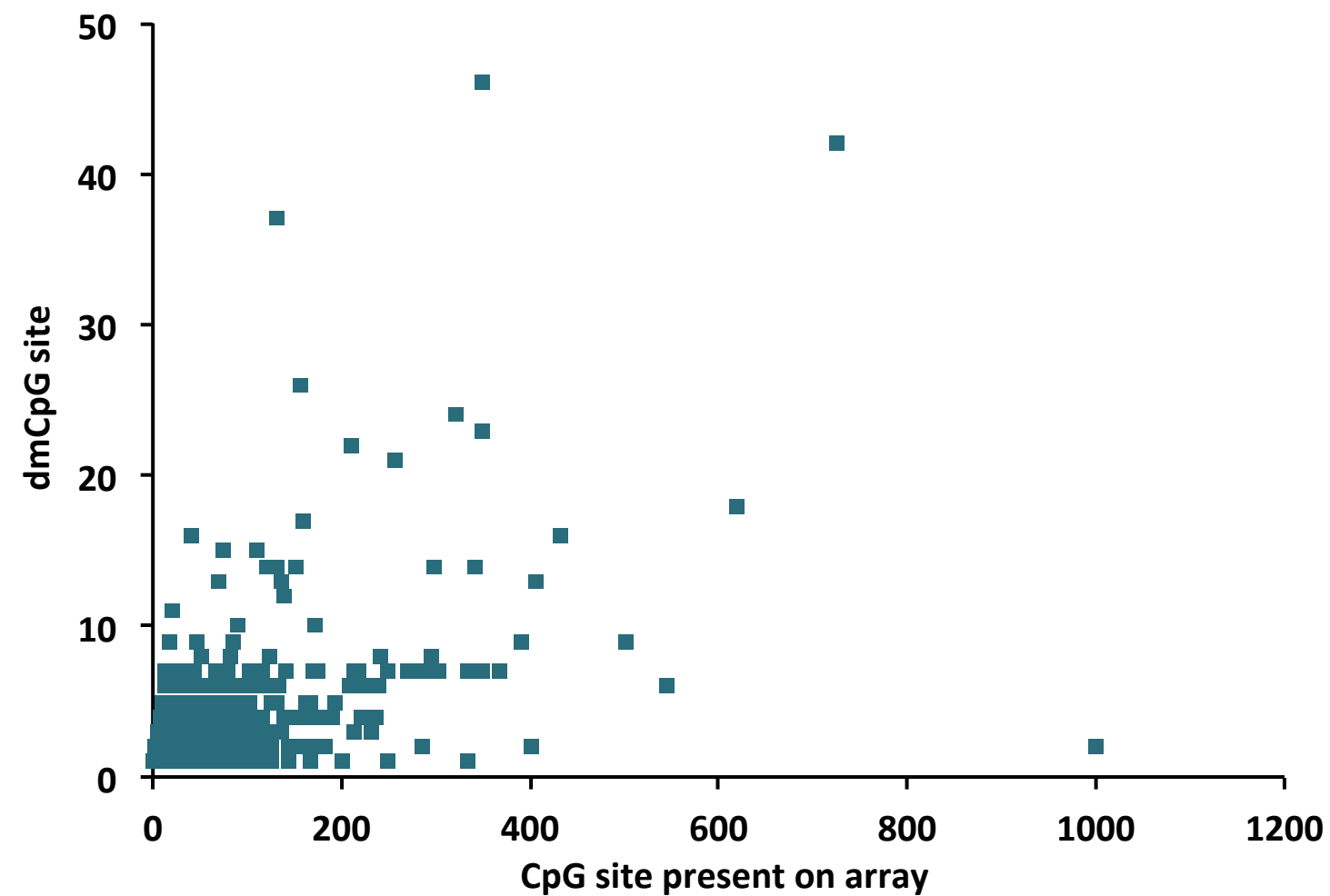

Supplement: Supplementary file 1 — Fig. S1 Numbers of CpGs present on array in a gene and dmCpG site for same gene. [file acel0013-0360-sd1.pdf]

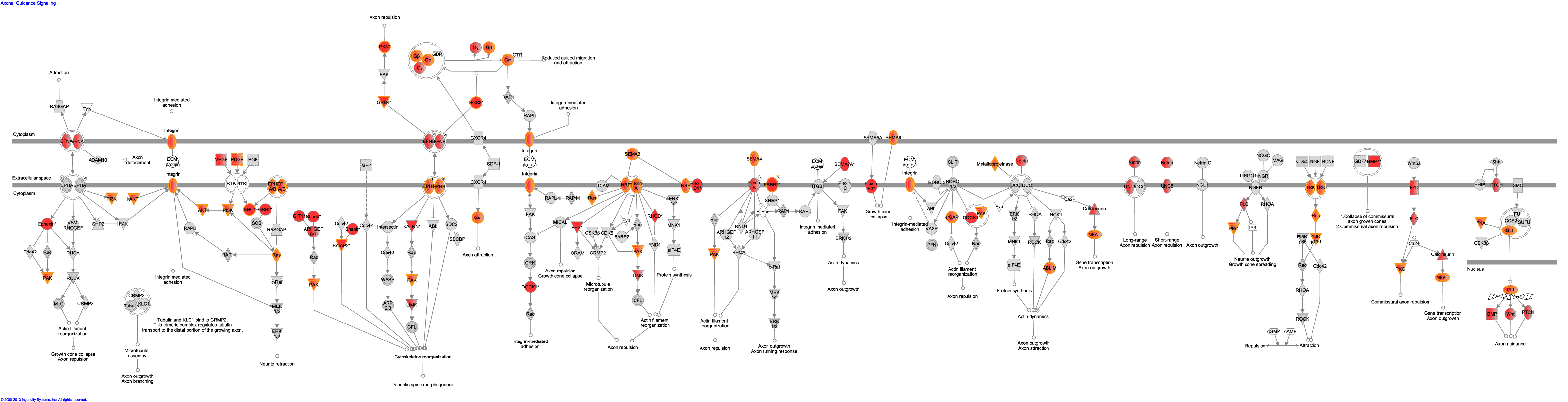

Supplement: Supplementary file 2 — Fig. S2 Ingenuity canonical axon guidance pathway. [file acel0013-0360-sd2.png]

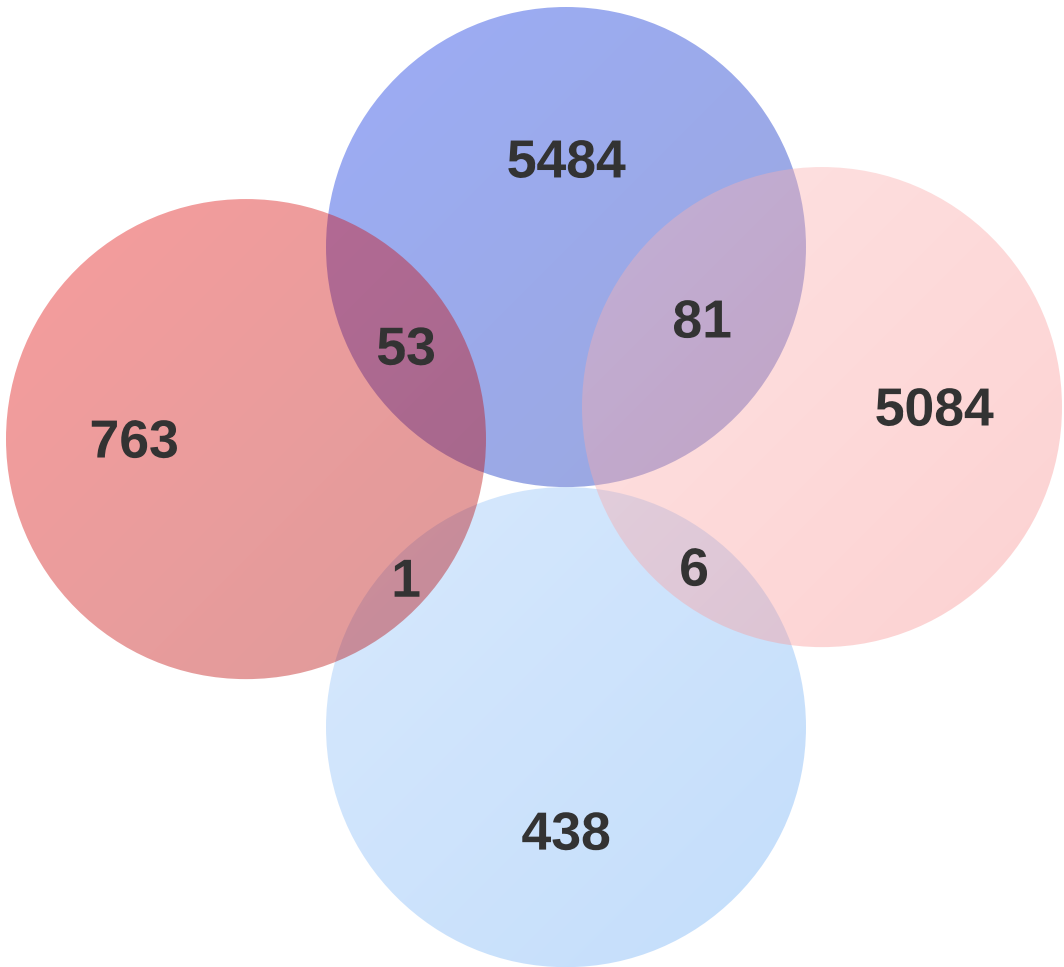

Supplement: Supplementary file 3 — Fig. S3 dmCpG sites that are common for this study and Heyn et al. [file acel0013-0360-sd3.pdf]
