# Supplementary material for: Genome-wide DNA methylation changes with age in disease-free human skeletal muscle
Source: Aging Cell. 2013 Dec 2;13(2):360–6. doi: 10.1111/acel.12180 (PMC3954952; doi:10.1111/acel.12180)
Supplement: Supplementary file 18 [file acel0013-0360-sd18.doc]

Supplement: Genome-wide DNA methylation changes with age in disease free human skeletal muscle

Supporting Information:

- Supplemental Methods and Citations
- Figure S1. Numbers of CpG’s present on array in a gene and dmCpG site for same gene.
- Figure S2. Ingenuity canonical axon guidance pathway.
- Figure S3. dmCpG sites that are common for this study and Heyn et al.
- Table S1. List of statistically significantly differentially methylated CpG probes with age, and raw Beta values for each subject (young and old).
- Table S2. Distribution of dmCpG.
- Table S3. ENCODE ChIP-Seq Significance Tool results.
- Table S4. Genes with at least one intragenic dmCpG site.
- Table S5. Ontology enrichment analysis.
- Table S6. Muscle specific canonical pathway analysis.
- Table S7. Differential methylation of axon guidance genes.
- Table S8. Correspondence of intragenic differential methylation to gene expression.
- Table S9. List of CpG sites concordant with age-group.
- Table S10. List of overlapping CpG predictors of biological age with Hannum et al. study.
- Table S11. Number of overlapping dmCpG sites with other studies.
- Table S12. Description of genomic regions.
- Table S13. Description of human subjects.
- Table S14. Illumina 450K data Quality Control Report

Supplemental Methods -

Human Subjects

Skeletal muscle biopsies from the vastus lateralis were taken from 24 healthy young (mean age 21 years old (yo), range 18-27 yo, SD 2) and 24 healthy older (mean age 73 yo, range 68-89 (yo), SD 5) male individuals using established procedures in our laboratory (Tarnopolsky et al.,2011). We used only males to maximize the likelihood of detecting changes in the epigenome of young and old tissue. None of the subjects were performing regular physical exercise and did not carry out any form of exercise in the 48 hours (h) preceding the biopsy, nor had they undergone an orthopedic procedure (i.e., joint arthroplasty) in the leg where the biopsy was taken. All subjects were studied between 0800h – 1200h biopsy in the fasted state and with no caffeine or alcohol intake for 24 h before the biopsy, and none of the subjects had significant medical disorders (diabetes, nerve or muscle disease, hypercholesterolemia requiring statins, cardiovascular disease (other than hypertension requiring at most one medication)) or smoked. Samples were taken under ethical approval from the Hamilton Health Sciences Research Ethics board (N = 32, IRB # 03-267, 05-376, 09-148) and the University of Oklahoma Health Sciences Center Institutional Review Board (N = 16, IRB # 13637) and all subjects provided informed, written consent. A detailed description of the human subjects is listed in Supplementary Table S13.

DNA extraction, quality control, and 450K chip processing & quantitation

DNA was extracted from muscle samples in a randomized fashion with regards to age using a Qiagen Qiacube DNA extraction robot. Extracted DNA was then subjected to quality control on an Agilent Bioanalyzer. DNA was labeled, hybridized, and scanned for the 450K DNA methylation chips as per the manufacturers instructions (Illumina). We stress that all samples were randomized for each chip, so batch effect potential was minimal. All hybridized chips QC passed criteria indicating complete bisulfite conversion etc. (Supplemental Table S14). The raw quantitated output files (ß values) were then exported as .csv files for further processing. The Illumina 450K DNA methylation array has been rigorously validated and proved to be consistent and robust in several studies (Dedeurwaerder et al.,2011;Pan et al.,2012;Roessler et al.,2012;Sandoval et al.,2011).

450K methylation analysis

We used the lumi/methylumi packages (Bioconductor, [www.bioconductor.org](http://www.bioconductor.org/)) to derive the quantile normalized values of methylation (M values) (Du et al., 2008) from the raw ß values. The lumi package is an accepted and validated method for determining differentially methylated sites using illumina arrays (Bock et al., 2012), and has been shown to perform better with M values than the raw ß values directly output from Genome Studio (Illumina) (Du et al., 2010). To derive sites of potential differential methylation (DM), we compared old versus young M values using a modified t-statistic (Smyth,2004), where defined DM sites based on statistical significance controlling false discovery rate (Benjamini & Hochberg,1995).

Concordant CpGs with age

For determining if select CpG sites were concordant with age group, we simply examine the consequences of different cut-offs measured on the continuous probes by constructing standard ROC curves, and measuring the area under the curve. Given that this process produced more than 20 probes with AUC = 1 (sites are excellent at distinguishing young from old) and up to 500 that nearly completely separated young from old, we did not try more complicated prediction algorithms. For this sample size, using another procedure, we cannot find a prediction model with better internally validated (cross-validated) prediction of age.

Within the data there a large number of sites where the ordering is “excellent” that is all the young samples are either smaller (or larger) than the corresponding old samples. Thus, any procedure, using cross-validation to estimate the misclassification rate, and considers using simple stepwise constant functions as candidates, will result in no misclassified samples. If one breaks the sample into any training and testing set, the resulting prediction function, based on a simple threshold (e.g., if biomarker X is > C then predict “old” where C is the maximum value of methylation of young samples in the training set) will result in perfect classification in the testing data set for many probes (see below). Once this is true, it means the estimated ability to predict future samples, based on data with the same data-generating mechanism as the sample, will be estimated to be “excellent”. However, such sites still have to be independently validated in another population.

Genome analysis of differentially methylated sites

GRCh37.p5 primary assembly was used for mapping probes to the genome. Coordinates of only REVIEWED and VALIDATED RefSeq genes were used in the analysis. A custom made perl script was made to locate CpG sites within various regions, such as intragenic or promoter regions. CpG island locations for hg19 was downloaded from R. Irizarry’s web page ([http://rafalab.jhsph.edu](http://rafalab.jhsph.edu/)). A CpG shore was defined as 2000 base pairs upstream and downstream of a CGI. The R package SkewR was used to locate GC skew coordinates with the GC_skew.hmm model (Ginno et al.,2012). Only those GC skew regions were used that overlap with a promoter by at least 1 nucleotide. Positive GC skew, named here as Gskew, is defined as a Gskew of >500 base pairs long, with at least 1 nucleotide overlap with the TSS or located on the gene side of the promoter, and co-oriented with the transcribed gene. Remaining GC skew that overlaps with a promoter is called GCskew.

The statistical significance for differentially methylated sites to be overrepresented or underrepresented in a region (for example, intragenic) relative to the expected distribution was calculated using standard tests of independent chi-square tests, where the null distribution was estimated via permutation (a low *p*-value indicating either significant over or under representation of dmCpG sites within the region compared to the proportion outside the region (Hope,1968)). To add further (conservative) restrictions for defining a region of interest, we calculated the odds ratio (OR - in this case equivalent to the ratios of probabilities of being within versus outside the region of interest) of a site being differentially methylated within the region, versus outside, and we also calculated the confidence interval (CI) and considered a region “interesting” if the CI excluded small magnitudes of the OR (between 1/1.25 and 1.25). We carried this out when CI’s were adjusted for multiple comparisons. Expected distribution is the fraction of dmCpG from all CpG sites for genome.

In order to compare changes in methylation and gene expression, we have identified suites of genes that have a minimum number of differentially methylated CpG sites (1, 2, 4, 8 and 16) within specific regions, for example, intragenic, 5’-end or CGITSS. If a gene has only hypermethylated or hypomethylated sites, it was designated as such. We then calculated the number of genes from either hypo- or hyper- methylated groups that overlap with over-expressed genes with age, as well as genes which we had characterized as decreased in expression abundance, and those that did not change expression level with age.

Ontology enrichment analysis

Ontology enrichment analysis was performed using the STOP tool (Wittkop et al.,2013) with the default options and using Benjamini-Hochberg multiple hypothesis correction and Entrez Gene identifiers. Following ontologies were used: Cell Cycle Ontology, Cell Type, Gene Ontology, Health Indicators, Human Disease Ontology, Pathway Ontology. For each foreground set (or input set) of genes we used a background that corresponds to probes available on the array chip. Enriched terms were called significant if the p-value is less than or equal to 0.01. For Ingenuity Pathway Analysis (Ingenuity Systems, [www.ingenuity.com](http://www.ingenuity.com/)) the list of all genes containing a intragenic dmCpG site and background genes were examined by Core IPA analysis examining both direct and indirect relationships. Only reference data which was specifically related to skeletal muscle that was experimentally validated or had a high predictive value was used. For enrichment analysis of bivalent chromatin domains, the same gene set (intragenic-2) as for ontology enrichment analysis was used. We determined the number of genes containing bivalent domains overlapping with our dmCpG probes compared with overlap of all array CpG probes. Hypergeometric test was used to determine significance of overrepresentation

Supplemental References

Benjamini Y., and Hochberg, Y.(1995).Controlling the false discovery rate: a practical and powerful approach to multiple testingJournal of the Royal Statistical Society. Series B (Methodological) 289-300.

Bock, C. (2012). Analysing and interpreting DNA methylation data. Nature

Reviews Genetics, 13(10), 705-19

Dedeurwaerder S., Defrance, M., Calonne, E., Denis, H., Sotiriou, C., and Fuks, F.(2011). Evaluation of the Infinium Methylation 450K technology.Epigenomics 3,771-84.

Du P., Kibbe, W.A., and Lin, S.M.(2008).lumi: a pipeline for processing Illumina microarray.Bioinformatics 24,1547-8.

Du, P., Zhang, X., Huang, C. C., Jafari, N., Kibbe, W. A., Hou, L., &

Lin, S. M. (2010). Comparison of beta-value and m-value methods for

quantifying methylation levels by microarray analysis. BMC

Bioinformatics, 11, 587.

Ginno P.A., Lott, P.L., Christensen, H.C., Korf, I., and Chédin, F.(2012).R-loop formation is a distinctive characteristic of unmethylated human CpG island promoters.Mol Cell 45,814-25.

Hope A.C.A.(1968).A simplified Monte Carlo significance test procedureJournal of the Royal Statistical Society. Series B (Methodological) 582-598.

Pan H., Chen, L., Dogra, S., Teh, A.L., Tan, J.H., Lim, Y.I., Lim, Y.C., Jin, S., Lee, Y.K., Ng, P.Y., Ong, M.L., Barton, S., Chong, Y.S., Meaney, M.J., Gluckman, P.D., Stunkel, W., Ding, C., and Holbrook, J.D.(2012).Measuring the methylome in clinical samples: improved processing of the Infinium Human Methylation450 BeadChip Array.Epigenetics 7,1173-87.

Roessler J., Ammerpohl, O., Gutwein, J., Hasemeier, B., Anwar, S.L., Kreipe, H., and Lehmann, U.(2012).Quantitative cross-validation and content analysis of the 450k DNA methylation array from Illumina, Inc.BMC Res Notes 5,210.

Sandoval J., Heyn, H., Moran, S., Serra-Musach, J., Pujana, M.A., Bibikova, M., and Esteller, M.(2011).Validation of a DNA methylation microarray for 450,000 CpG sites in the human genome.Epigenetics 6,692-702.

Smyth G.K.(2004).Linear models and empirical bayes methods for assessing differential expression in microarray experiments.Stat Appl Genet Mol Biol 3,Article3.

Tarnopolsky M.A., Pearce, E., Smith, K., and Lach, B.(2011).Suction-modified Bergström muscle biopsy technique: experience with 13,500 procedures.Muscle Nerve 43,717-25.

Wittkop T., TerAvest, E., Evani, U.S., Fleisch, K.M., Berman, A.E., Powell, C., Shah, N.H., and Mooney, S.D.(2013).STOP using just GO: a multi-ontology hypothesis generation tool for high throughput experimentation.BMC Bioinformatics 14,53.

Supplemental Figures -

Fig S1 -


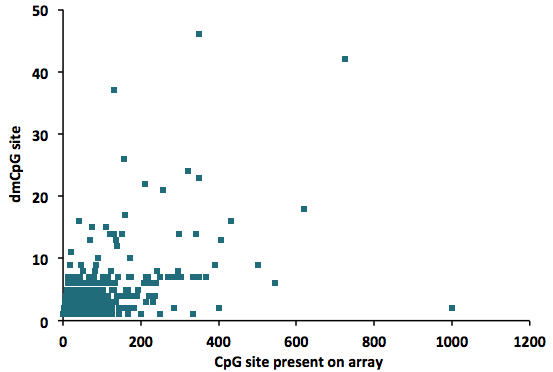


Supplementary Figure S1 - Numbers of CpG’s present on array in a gene and dmCpG site for same gene. Y-axis - number of dmCpG sites for a given gene; X-axis - number of CpG sites present on microarray chip for the same gene.

Fig S2 -


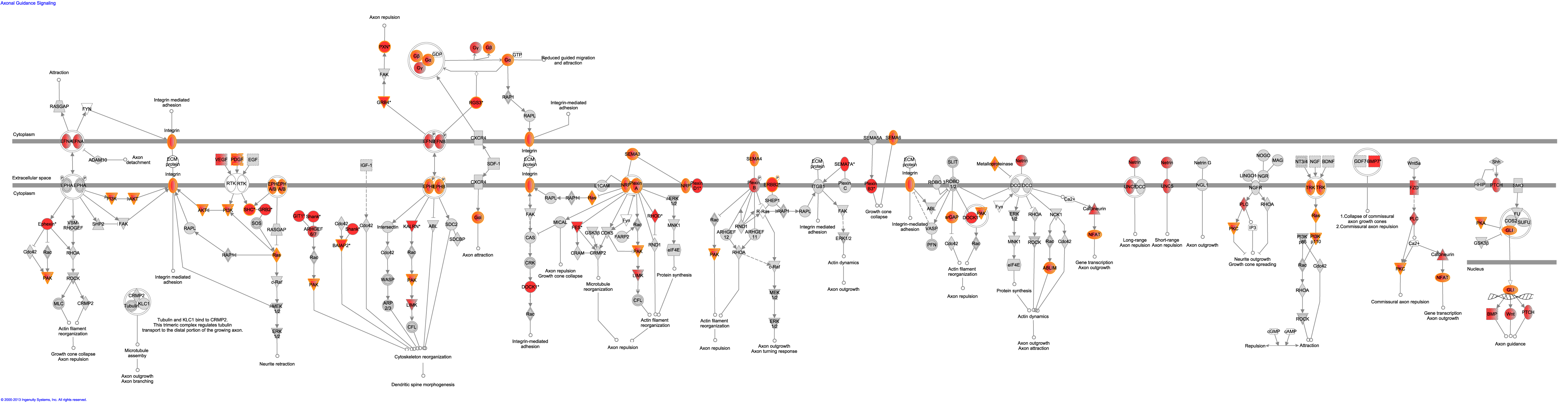


Supplementary Figure S2 - Ingenuity canonical axon guidance pathway. Diagram of the “axon guidance signaling” pathway generated in IPA software. The red nodes indicate genes with differentially methylation in the intragenic region. Clear nodes were unaffected in this dataset.

Fig S3 -


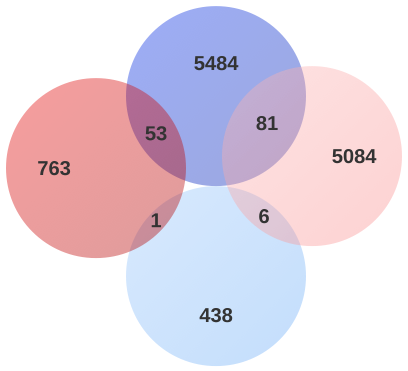


Supplementary Figure S3 - dmCpG sites that are common for this study and Heyn et al. Blue circles - this study; red - Heyn et al. Dark color - hypermethylated dmCpG sites with age; light color - hypomethylated dmCpG sites.

Supplementary Table S1 - List of differentially methylated CpG probes with age.

Supplementary Table S2 - Distribution of dmCpG. Distribution of dmCpG, hypermethylated dmCpG and hypomethylated dmCpG in different genomic regions. Detailed region description can be found in Supplementary Table S12. Column abbreviation: all probes - number of all CpG probes available on array and located in a region; probe - number of dmCpG; fraction - fraction of dmCpG from all CpG for a region; logOvsE - log2 ratio of observed (fraction of dmCpG in a region) vs expected (fraction of dmCpG in genome); or - odds ratio of a site being differentially methylated within the region, versus outside the region; lowerci - lower confidence interval; upperci - upper confidence interval; interesting - TRUE means significant, if the CI excluded small magnitudes of the OR (between 1/1.25 and 1.25); FALSE - not significant.

Supplementary Table S3 - ENCODE ChIP-Seq Significance Tool results.

Supplementary Table S4 - Genes with at least one intragenic dmCpG site. Number of intragenic, intragenichyper and intragenic-hypo differentially methylated CpG sites.

Supplementary Table S5 - Ontology enrichment analysis. Columns: categories, see description in Supplementary Table 12; study count, number of genes in foreground associated with the term; study total, total number of genes associated with the term; background count, number of genes from background associated with the term; background total, total number of genes associated with the term.

Supplemental Table S6 - Muscle specific canonical pathway analysis. Using Ingenuity Pathway Analysis the genes with at least one intragenic dmCpG site (see Supplemental Table 3) were analyzed limiting the focus to muscle specific data. Canonical pathway analysis reconfirmed the presence of “axon guidance signaling” as the most over-represented pathway in this dataset. This table includes the methylation status the of dmCpG sites in the members of this pathway.

Supplemental Table S7 - Differential methylation of axon guidance genes. Genes containing dmCpG sites within the intragenic region which are members of the canonical “axon guidance signaling” pathway. Each gene is shown with the number of differentially methylated probes and their methylation status.

Supplementary Table S8 - Correspondence of intragenic differential methylation to gene expression. The number of genes that have a minimal number of dmCpG with age and did not change expression, increased expression or decreased expression with aging. Columns: meth: number of genes with a minimal dmCpG; up - number of genes from methylation group that increased gene expression with age; down - number of genes from methylation group that decreased gene expression with age; unchanged - number of genes from methylation group that unchanged gene expression with age. Categories: see description in Supplementary Table S12; number at the end is the minimal number of dmCpG; if a category is labeled with hyper- or hypo- suffixes, this indicates that the gene has only hypermethylated or hypomethylated dmCpG.

Supplementary Table S9 - List of CpG predictors of biological age.

Supplementary Table S10 - List of overlapping CpG predictors of biological age with Hannum et al. study.

Supplementary Table S11 - Number of overlapping dmCpG sites with other studies. Number of dmCpG sites from other studies, and number of common dmCpG sites between studies.

Supplementary Table S12 - Description of genomic regions. Biotype abbreviation/description could be found at Genbank web site.

Supplementary Table S13 - Description of human subjects.

Supplementary Table S14 – Illumina 450K array quality control measures.
